# Metagenomic airborne resistome from urban hot spots through the One Health lens

**DOI:** 10.1111/1758-2229.13306

**Published:** 2024-06-23

**Authors:** Lucia Maestre‐Carballa, Vicente Navarro‐López, Manuel Martinez‐Garcia

**Affiliations:** ^1^ Department of Physiology, Genetics, and Microbiology University of Alicante Alicante Spain; ^2^ Instituto Multidisciplinar Para el Estudio del Medio Ramon Margalef University of Alicante Alicante Spain; ^3^ Clinical Microbiology and Infectious Disease Unit Hospital Universitario Vinalopó Elche Spain

## Abstract

Human activities are a significant contributor to the spread of antibiotic resistance genes (ARGs), which pose a serious threat to human health. These ARGs can be transmitted through various pathways, including air, within the context of One Health. This study used metagenomics to monitor the resistomes in urban air from two critical locations: a wastewater treatment plant and a hospital, both indoor and outdoor. The presence of cell‐like structures was confirmed through fluorescence microscopy. The metagenomic analysis revealed a wide variety of ARGs and a high diversity of antibiotic‐resistant bacteria in the airborne particles collected. The wastewater treatment plant showed higher relative abundances with 32 ARG hits per Gb and m^3^, followed by the main entrance of the hospital (indoor) with ≈5 ARG hits per Gb and m^3^. The hospital entrance exhibited the highest ARG richness, with a total of 152 different ARGs classified into nine categories of antibiotic resistance. Common commensal and pathogenic bacteria carrying ARGs, such as *Moraxella*, *Staphylococcus* and *Micrococcus*, were detected in the indoor airborne particles of the hospital. Interestingly, no ARGs were shared among all the samples analysed, indicating a highly variable dynamic of airborne resistomes. Furthermore, the study found no ARGs in the airborne viral fractions analysed, suggesting that airborne viruses play a negligible role in the dissemination of ARGs.

## Graphical abstract

The spread of antibiotic resistance genes (ARGs) through various pathways, including air, presents a significant risk to human health. This study's air microbiology analysis revealed a high relative abundance and a wide variety of ARGs, as well as a diverse range of antibiotic‐resistant bacteria in airborne particles collected from urban hot spots. These hot spots include hospitals and wastewater treatment plants. The findings underscore the critical need for ongoing surveillance and intervention strategies to mitigate this public health threat.

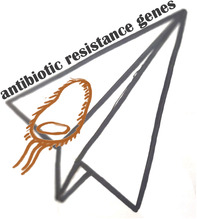

## INTRODUCTION

One of the most noteworthy accomplishments in medical history is the advent of antibiotics. Nevertheless, the rise and dispersion of antibiotic‐resistant bacteria poses a challenge to global public health (Neu, 1992), and indeed by 2050, an annual mortality toll of 10 million people has been predicted (Balouiri et al., 2016). Different antibiotic resistance mechanisms have been described that are inherent to microorganisms or can be acquired genetically by horizontal gene transfer (Reygaert, 2018). In contrast to other environments, the diversity and relative abundance of ARGs in the atmosphere are understudied (Li et al., 2018; Zhou et al., 2023). Airborne microbes potentially carrying ARG can be easily inhaled by humans reaching mucous membranes in the respiratory system and the oral cavity. In particular, antibiotic‐resistant bacteria associated with airborne fine particulate matter exacerbate this health issue because these particles can penetrate deeply into the alveolar region and even enter the bloodstream (Jin et al., 2017; Li et al., 2019). Inhaled ARGs have been found to expose human beings to a concentration of 10^2–3^ copies/m^3^‐air (Xie et al., 2018), and their pathogenic bacteria hosts could increase the chances of resistant infections through air inhalation. Considering that an adult can breathe more than 10,000 L of air per day (Mack et al., 2019), the indoor and outdoor surrounding atmosphere could represent a significant exposure pathway to ARGs.

Airborne ARGs abundance and biogeography exhibit a profound dissimilarity and are highly dynamic. For instance, the ARG abundances were detected to vary nearly 100‐fold between cities like Bandung (Indonesia) and San Francisco (USA) (Li et al., 2018). The emission of airborne particles from terrestrial sources, such as waste landfills, treatment systems and animal farms, play an important role in the dispersion of airborne ARGs (Bai et al., 2022; Li et al., 2020). Later, air flow clouds, precipitation, and snowfall contribute to the transmission of ARGs across various environments (Zhu et al., 2021). In many of these studies, quantitative PCR targeting multiple genes—up to hundreds of different ARGs—has been successfully employed to unveil the resistome in airborne particles (Zhou et al., 2023). Additionally, metagenomics, which can sequence nearly the whole genetic information from microbes in a sample, is another powerful approach to unravelling the intricate dynamics of airborne resistomes.

Here, through the use of metagenomics, we aim to investigate the ARG profiles and resistomes present in the air of two of the most common hot spots of ARG emission within a city scale: a hospital and a wastewater treatment plant (WWTP). Our study represents a comparison of both types of air samples using the same methodology including sampling, metagenomics and bioinformatic analysis, which in the case of air microbiology would reduce the existing bias and variability when comparing similar samples obtained from other studies with different approaches. These hot spots represent important sources of potential emission of ARGs and antibiotic‐resistant bacteria, whose study addresses one of the health challenges included in the One Health perspective, antimicrobial resistance, which affects the health of people, animals and ecosystems since they are tightly associated and interdependent (Adisasmito et al., 2022).

## EXPERIMENTAL PROCEDURES

### Sampling sites

To determine the ARGs profile in Alicante's air, the air of different ARGs hot spots of the city of Alicante (331,000 citizens) was sampled: (1) the roof of the largest hospital in Alicante city (near the ventilation outlets of a re‐opened building 2 weeks before sampling at the Hospital General Universitario Dr. Balmis, 38°21′47.9″ N, 0°29′08.6″ W; date 14 July 2020), (2) main entrance or reception of the hospital (22 October 2019), (3) in situ sampling in the vicinity of the biodigester and bioreactor at the WWTP of the city of Alicante (named l'Alacantí Nord), which receives municipal wastewater (38°25′30.8″ N, 0°25′10.1″ W; date 27 June 2020), and two more spots located out of that WWTP but nearby (≈500 m) named as WWTP out 1 (11 July 2020) and WWTP out 2 (11 July 2020).

### Air sample processing and sequencing

**FIGURE A1:**
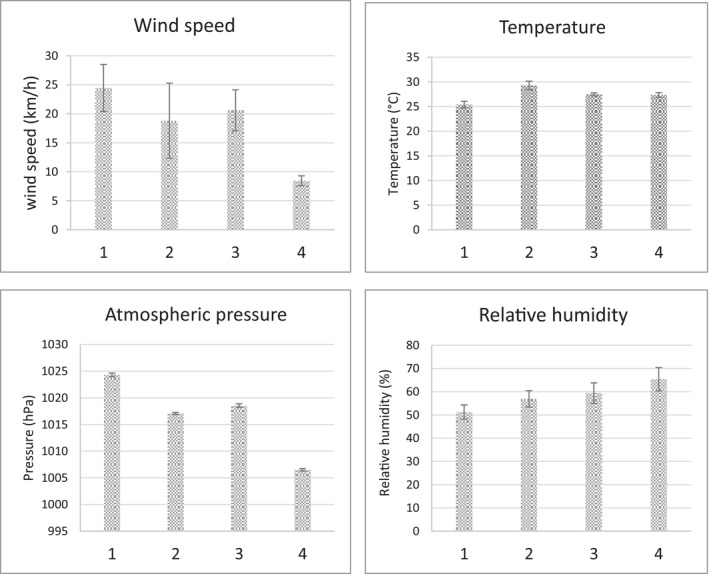
Environmental parameters measured for each sample. Mean and standard deviation for the wind speed (km/h), temperature (°C), atmospheric pressure (hPa) and relative humidity (%) measured while sampling (3–6 h). The data were provided by AVAMET (https://www.avamet.org/). For the Hospital sample, the station used was Rabassa (Alicante; 38.37, −0.4999; 14 July 2020), and for the WWTP samples, the station was placed at the aerodrome of Mutxamel (Alicante; 38.44077, −0.474172; 27 May 2020, 11 July 2020 and 12 July 2020). The instrument used was Davis Vantage Pro2 (Davis instruments, USA). WWTP, wastewater treatment plant.

Outdoor temperature, humidity, atmospheric pressure and wind speed data were provided by the official meteorological agency AVAMET (https://www.avamet.org; for details Figure A1). Air samples were taken with a Coriolis Micro (Bertin Technologies) in 10 mL of sterile PBS 1X, at a flow rate of 300 L/min and 0.5 m above the ground. Prior to sampling, different parts of the air sampler in contact with air and sampling buffer were autoclaved (e.g., air inlet tube and buffer receptacle). The total air volume sampled ranged from 52.5 to 108 m^3^ (Table S1). As a negative control, a similar volume of air (68 m^3^) was sampled inside a laminar flow hood in our laboratory and processed identically to the rest of the samples to monitor potential DNA contamination from manipulation, reagents, metagenomic DNA library prep or DNA sequencing. During air sampling, as commonly described for air samplers (Behzad et al., 2015) and to prevent evaporation of sampling buffer, sterile PBS 1X was added up to 10 mL volume each 10 min. After air sampling, the 10 mL PBS 1X buffer containing airborne particles and microorganisms was transported in ice to the laboratory and immediately processed for nucleic acid extraction and SYBR Gold staining. For nucleic acid extractions, samples were filtered through 0.2 μm Isopore Membrane Filters (Millipore, Ref. GTTP02500). Furthermore, the elute from the 0.2 μm filters from the roof hospital sample and the in situ sample collected at the WWTP, was filtered again through 0.02 μm Anodisc filters (Whatman, GE Healthcare Life Sciences, Ref. 6809–6002) to retain nanoparticles (e.g., viruses). All filters were stored at −80°C until use. Nucleic acids were extracted from filters using a DNAeasy PowerSoil Pro kit (Qiagen, Ref. 47014) following the manufacturer's protocol. DNA concentration was measured using a fluorimeter instrument Qubit with an HS dsDNA Qubit kit (Thermo Fisher Scientific, Ref. Q32851; detection limit: 0.01 ng/μL). Due to the very low input of extracted DNA, metagenomic libraries were performed strictly following the Nextera XT protocol recommended for ultra‐low biomass samples that were published by Rinke et al. (2016). For this step, as recommended in that study, a metagenomic library prep control sample was included and comprised of lambda phage DNA (500 μg/mL; BioLabs, Ref. N3011S) that was processed as the rest of the samples and used for cleaning up the sequencing data for removing potential contaminant reads from exogenous DNA (Rinke et al., 2016). A whole genome amplification by multiple‐displacement amplification (MDA) was applied to the samples extracted from the 0.02 μm Anodisc filters since the amount of extracted DNA precluded the standard metagenomic library preparation. MDA was also applied for the >0.2 μm roof sample. MDA was performed as a described method in the following references (De La Cruz Peña et al., 2018; Martinez‐Hernandez et al., 2017; Rinke et al., 2016). As a negative control during MDA, sterile MQ water was included. All samples were sequenced using Illumina Seq X (pair‐end, 150 bp) in Macrogen facilities (Seoul, South Korea).

For visualisation of microbes and particles, the in situ WWTP air sample (54 m^3^; 7 mL; 11 October 2019) was filtered through 0.02 μm Anodisc filters (Whatman, GE Healthcare Life Sciences, Ref. 6809–6002), stained for 15 min with 0.5 μL SYBR Gold 10× (ThermoFisher Scientific, Ref. S11494) combined with 19.5 μL of MQ water, then washed with MQ water (0.2 μm‐filtered) three times. Once the filter was dry, it was mounted using citifluor AF1 (Electron Microscopy Sciences, Ref. 17970–100) and later inspected under a Leica epifluorescence microscope SP2.

### Bioinformatic analysis

Raw data from air samples was quality‐filtered using Trimmomatic 0.36 (Bolger et al., 2014) (SLIDINGWINDOW:4:20, MINLEN:36). Then, sequenced samples and blank controls (see above) from air sampling were compared to remove potential contaminant reads from exogenous DNA. For that, reads in samples that matched with blank controls (query coverage ≥50% and identity ≥80%) were removed from the analysis. Taxonomic classification of quality filtered reads was performed with the Kaiju program (Menzel et al., 2016) using nr_euk (22 December 2023), which contains sequences for Archaea, Bacteria, viruses, fungi, and microbial eukaryotes from the NCBI RefSeq non‐redundant protein collection. A subset from the reads (1%–2%) that remained unclassified by Kaiju was compared with the nt database (NCBI; e‐value <10^−5^, identity ≥95%) for their taxonomical annotation.

The filtered and clean reads were assembled using SPAdes (Bankevich et al., 2012) using the ‘‐meta’ option, and only the obtained contigs >500 bp were considered for further analysis. From those contigs, ORFs were predicted using Prodigal (Hyatt et al., 2010). ARGs were annotated by comparing the ARG databases ARG_ANNOT (Gupta et al., 2014), RESFAMS (Gibson et al., 2015), and CARD (Jia et al., 2017) with both the assembled (ORFs) and unassembled (reads) data using blast (Maestre‐Carballa et al., 2019, 2022). Only the best hits with a bit‐score ≥ 70, e‐value <10^−5^, and identities ≥50% or ≥ 90% (both thresholds were initially compared selecting later the cut‐off of ≥90% as likely the most reliable) were considered as potential ARG. The housekeeping genes present in the used ARGs databases were not considered in our metagenomic analysis due to the difficulty of determining if they were bona fide ARGs using our thresholds (Maestre‐Carballa et al., 2022) According to the database CARD (Jia et al., 2017) or the nr database (NCBI), the detected ARG were grouped by the antibiotic they confer resistance to. ARG abundance and normalisation were estimated by dividing the total number of ARGs per Gb of metagenome (assembled or unassembled) and if necessary, by volume sample as well. Estimation of shared ARGs in the analysed samples was carried out using a Venn diagram from the bioinformatics UGent webpage (https://bioinformatics.psb.ugent.be/cgi-bin/liste/Venn/calculate_venn.htpl). In addition, to confirm that the detected ARGs were present in prokaryotic contigs, a further bioinformatic step was considered to detect potential viral contigs that might be present in the prokaryotic assembled metagenomes. For that, all assembled metagenomes were analysed with VirSorter2 (Guo et al., 2021), which identified viral contigs (max. score ≥0.9). Contigs without detected viral proteins were therefore confirmed to be of prokaryote origin. The contigs with at least one ARG were annotated using Kaiju (Menzel et al., 2016) comparing them with the database nr_euk (22 December 2023).

## RESULTS AND DISCUSSION

### Sampling, DNA extraction and fluorescence microscopy of airborne particles

**FIGURE 1:**
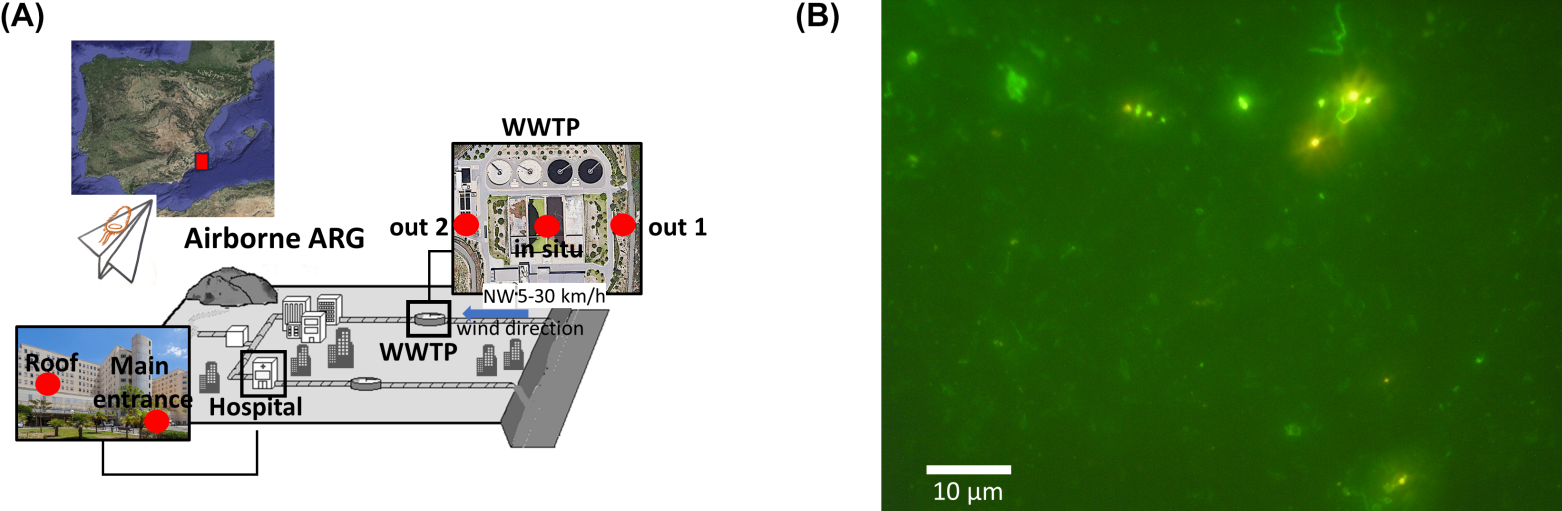
Airborne antibiotic resistance genes from urban hotspots. Five air samples were obtained using a Coriolis collector to analyse the urban airborne resistome: two from the General Hospital of Alicante (roof and main entrance) and three from the WWTP L'Alacantí Nord (out1, out2 and in situ) (A). An air sample from the WWTP in situ was filtered (0.2 μm) and then stained with SYBR Gold to prove the presence of microorganisms captured with Coriolis μ air sampler (B). ARGs, antibiotic resistance genes; WWTP, wastewater treatment plant.

There is a growing concern about urban air quality because of the potential dispersion of ARGs throughout airborne particles. In this study, two urban hot spots of ARG emission (a hospital and WWTP in Alicante city, Spain), were selected and subjected to airborne ARG detection by metagenomics (Figure 1A). A total of five samples were taken and analysed: three outdoor samples from one of the largest WWTP in Alicante city (≈331,000 citizens) and two samples from the largest hospital in this city (Figure 1A). Sampling of air from the different locations (≈50–100 m^3^ of air was sampled, Table S1) was performed in situ using a Coriolis micro instrument according to the user guide (Bertin Technologies, France). In addition, the potential resistome in the viral fraction (size range between 0.2 and 0.02 μm; viral particles associated with droplets, aerosols, or other particles of a size that enables their capture by Coriolis microsampling) was addressed for the hospital roof sample and the in situ sample taken at the WWTP. Furthermore, to monitor potential DNA contamination in our study, we carefully included metagenomic blanks (see methods for details), and a similar volume of air (≈70 m^3^) was sampled inside a laminar flow hood in our laboratory and processed identically to the rest of samples.

First, SYBR Gold dye, which stains mainly DNA, was used to prove the presence of microorganisms in the air samples. As shown in Figure 1B, a diversity of small and large cell‐like structures were observed within the range of typical size of microbes. In addition, other very large structures that do not resemble prokaryotic cells were also detected. Once, we confirmed the presence of cell‐like structures, total microbial DNA was extracted yielding 0.01–0.2 ng of DNA/m^3^ of air (Table S1). DNA was not detected in the blank sample (air collected in the laminar hood) indicating that our procedure and buffers used during sampling were free of DNA and contamination. The highest concentration of DNA was obtained for one of the samples collected near the WWTP (named WWTP out 2), while the lowest was obtained for the outdoor sample collected on the hospital roof (0.014 ng/m^3^) suggesting that the total microbial load released in airborne particles from the air circulation system of the hospital was negligent. Those obtained values were similar to those found on the roof of a Hospital in San Diego (USA), yielding 0.016 ng/m^3^ (King et al., 2016). In contrast, the DNA extracted from the air of a Chinese hospital rooftop had a higher concentration (≈0.04 ng/m^3^; 1440 m^3^ sampled) (Wu et al., 2022), which could be due to the different sampling methods used. In addition, we speculate that the lower amount of DNA in airborne particles in our data from the hospital roof could be influenced by the recent reconstruction and public opening of the building (2 weeks prior to the sampling day). The indoor sample taken at the main entrance of the hospital remarkably showed a similar amount of DNA in airborne particles (0.07 ng of DNA/m^3^ of air) than that sample collected in situ at the WWTP close to the biodigesters (Table S1).

Regarding the viral fractions putatively present in airborne particles collected from the hospital roof and the WWTP within the range size of 0.2–0.02 μm, no DNA was detected suggesting that the viral load in these obtained fractions was very low. Similarly, for the prokaryotic fraction of the outdoor sample collected on the roof of the hospital, DNA was not detected until it was concentrated, obtaining an initial value of 0.0096 ng/μl below the minimal threshold Qubit limit (0.01 ng/μL). To overcome that and allow metagenomic sequencing, these samples were subjected to whole‐genome amplification prior to metagenomic libraries, which is a method that can detect and amplify as little as a single viral genome present in a sample (De La Cruz Peña et al., 2018; Martínez Martínez et al., 2020; Martinez‐Hernandez et al., 2017). It is important to remark, that this type of amplification distorts the original proportions of the DNA templates (Yilmaz et al., 2010).

### Taxonomic composition of microorganisms in airborne particles

**FIGURE 2:**
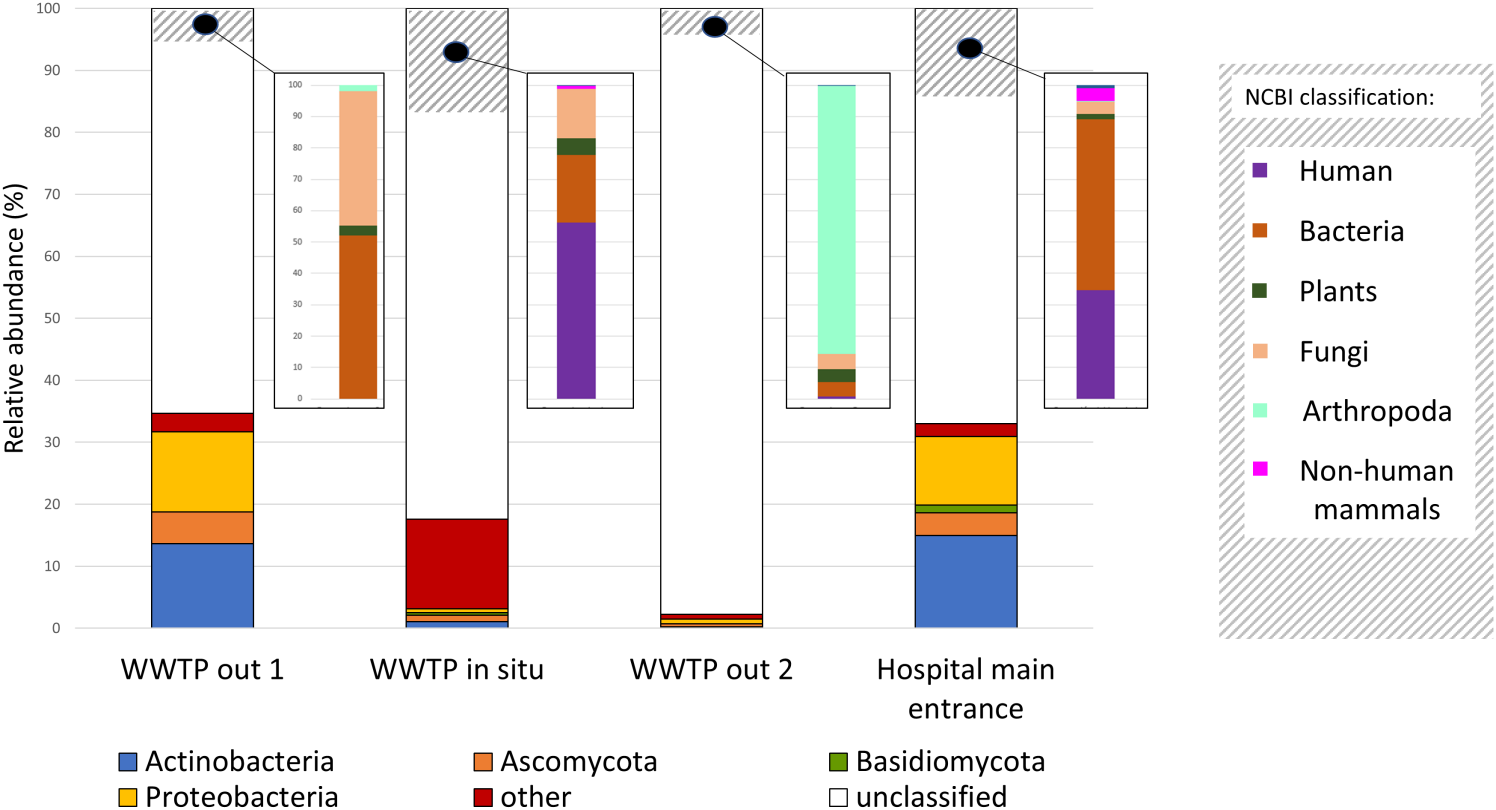
Metagenomic identification of microorganisms and organisms transported by airborne particles. Quality‐filtered metagenomic reads were compared for taxonomic identification with Kaiju program. The subset of the reads that remained unclassified (depicted as white) with Kaiju underwent further comparison with the NCBI databases. The proportion of reads unclassified by Kaiju but classified by NCBI are represented through a grey striped pattern. WWTP, wastewater treatment plant.

Metagenomics was used to assess the overall taxonomic identity of airborne microorganisms in the analysed samples irrespective of whether they carried ARGs. For that, we used quality‐filtered unassembled metagenomic data that were analysed with Kaiju and BLAST programs (see details in Methods). As the utilisation of MDA introduces biases that compromise the accuracy of abundance results (Yilmaz et al., 2010), we have chosen not to present the abundance data in this study for the roof and <0.2 μm samples to ensure the integrity and reliability of our findings. As shown in Figure 2, not only prokaryotes were collected, but also genetic information from other organisms was obtained, such as humans, other non‐human mammals, plants, fungi and arthropods. Despite computational efforts being carried out using different comprehensive databases from NCBI, a large fraction of metagenomes remained unclassified or the classification was unclear (63%–98%). Regarding the microorganisms classified, bacterial taxa were the dominant ones in all air samples, as observed in other studies (Cao et al., 2014). For the air samples collected in situ or nearby WWTP, as shown in Table S2, the bacterial species richness was high since we obtained taxonomic assignment of reads to more than 1000 different bacterial species. The most abundant airborne bacteria in these samples were common human commensals and pathogens, in addition to genera from other sources, such as soil like *Sinorhizobium* or *Frankiales* (Lie et al., 1984) or bacteria typical of wastewater systems, such as *Escherichia coli* (Yu et al., 2022) (Table S2).

The dominant taxa found in l'Alacanti Nord WWTP air, such as *Actinobacteria, Firmicutes, Proteobacteria* and *Bacteroidetes*, were also frequent in other studies of airborne particles in different WWTPs carried out in Beijing (Han et al., 2019) or the Hunan Province (Zeng et al., 2022), even though the relative abundance proportions differ. The main fungi phyla found were *Ascomycota* and *Basidiomycota*, which are known to release spores into the atmosphere and are usually found in air samples (Després et al., 2007).

For the main entrance of the hospital, reads assigned to common skin microbes, such as *Micrococcus, Kocuria, Streptococcus, Cutibacterium* or fungi *Malasezzia* (Loomis et al., 2021; Myers et al., 2023), were obtained. For the roof sample, we identified that the majority of the species (98%) were also present at the main entrance. However, as previously discussed, the application of MDA could alter the original proportions of DNA templates, preventing us from drawing any conclusions regarding accurate taxonomic relative abundance.

### Abundance of antibiotic‐resistance genes in airborne particles

**FIGURE 3:**
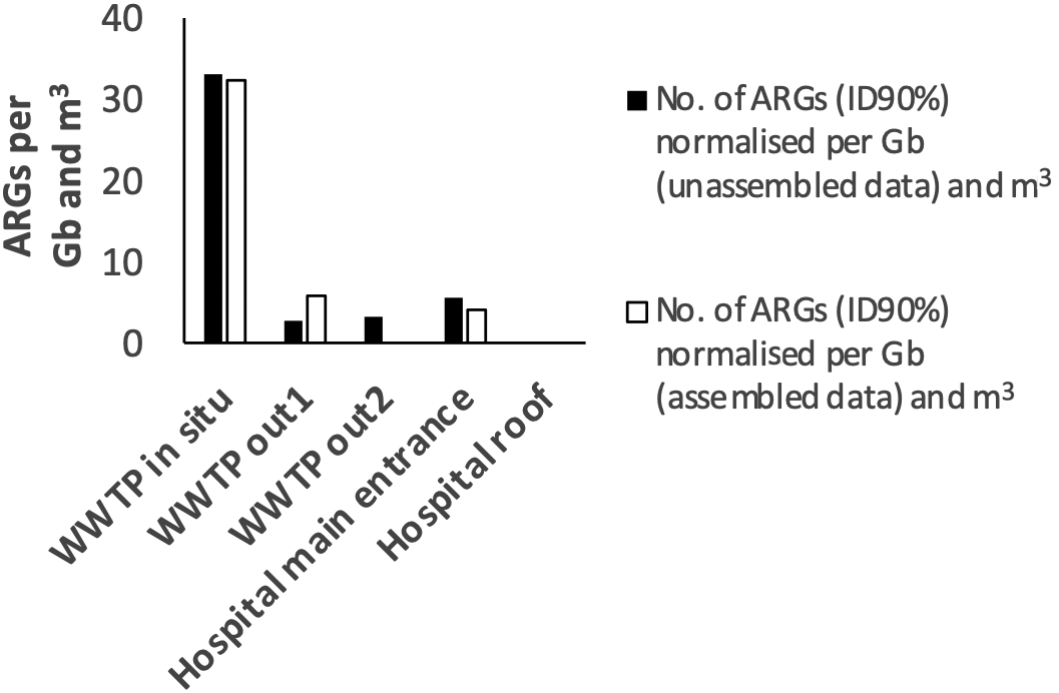
Metagenomic relative abundance of antibiotic resistance genes. Relative abundance of ARGs was carried on for both the assembled and unassembled metagenomic air data, with being the air sample WWTP input the one with the highest ARGs abundance. No ARG was detected in the analysed viral fractions of the collected airborne particles nor hospital prokaryotic roof sample. ARGs, antibiotic resistance genes; WWTP, wastewater treatment plant.

**FIGURE A2:**
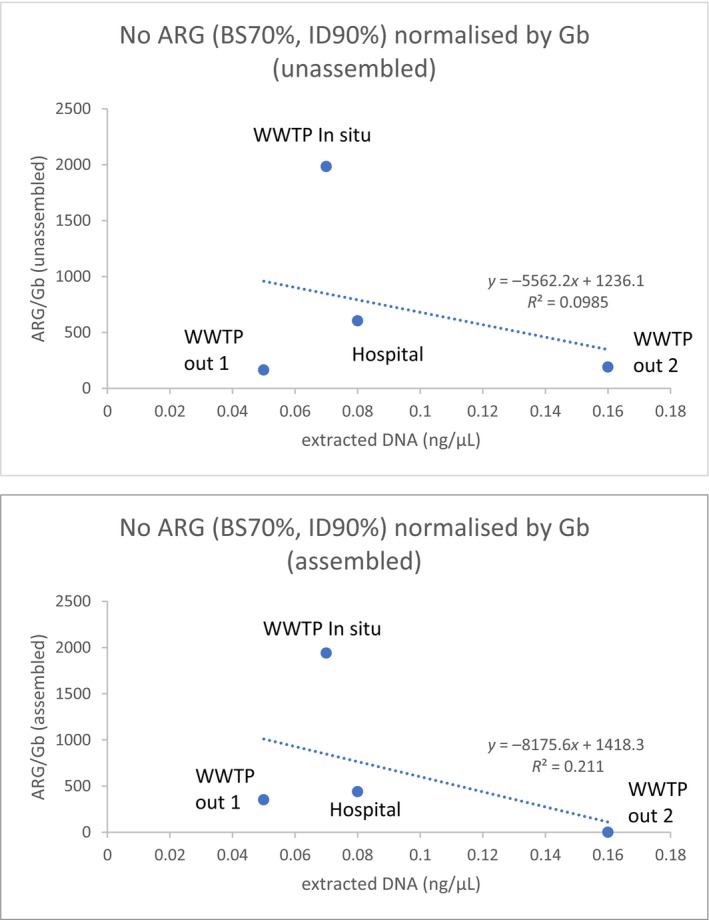
Comparison of the DNA extracted (ng/μL) with the antibiotic resistance genes found per Gb for unassembled and assembled data. We did not find a relation between the amount of DNA extracted and the abundance of antibiotic resistance genes found neither in assembled nor in unassembled data (expressed as Gb). ARGs, antibiotic resistance genes; WWTP, wastewater treatment plant.

Estimation of relative abundance of ARGs in metagenomes can be performed using unassembled or/and assembled data (Maestre‐Carballa et al., 2019). Each approach has some advantages and inconveniences. For instance, in general, a significant fraction of sequenced metagenomes remains unassembled, and often struggle to assemble genome fragments from abundant and diverse bacteria present in the sample (Martínez Martínez et al., 2020; Martinez‐Hernandez et al., 2017; Ramos‐Barbero et al., 2019). In contrast, addressing assembled data allows us to confidently identify and study ARG within the genomic context (Maestre‐Carballa et al., 2022; Zhang et al., 2022). Here, we sought to include both approaches that are complementary to analyse the resistome of the analysed air samples. The relative abundance of ARG was higher in the sample taken in situ at the WWTP next to the biological bioreactor, with approximately 32 total ARG hits per Gb of metagenomic data and m^3^ of air, which was followed by WWTP out1 (sample taken near the WWTP) and the hospital main entrance that showed ≈5 total ARG hits per Gb of metagenomic data and m^3^ of air (Figure 3). The higher abundance of ARGs in WWTP compared to that collected from the Hospital obtained in our study was also found in other independent study comparing metagenomic abundances of air samples in China, where for example the abundance of ARG in the air's hospital in April (He et al., 2020) was lower than the abundance obtained in the studied WWTP (Yang et al., 2018) carried out in the same month. We did not find a correlation (*R* ^2^ < 0.22) between the extracted DNA and the ARGs found for both unassembled and assembled data (Figure A2). No ARGs were detected in the prokaryotic fraction (>0.2 μm) of the hospital roof sample. For the putative viral fractions collected outdoors from the hospital roof and in situ at the WWTP, no ARG was detected with the employed criteria (see methods for more details). It is important to remark that our data suggest that the dissemination of ARGs throughout airborne viral particles is negligent since no ARG was detected in the analysed viral fractions from the WWTP and hospital. While our analysis focused only on viruses that could be collected by Coriolis micro sampling instrument (e.g., viruses associated with droplets, aerosols or particles >0.5 μm), which struggle to recover suspended individual viral particles not associated with particles or aerosols, our data align with other metagenomics results indicating that viruses are unlikely ARG carriers (Enault et al., 2017; Maestre‐Carballa et al., 2019).

**FIGURE A3:**
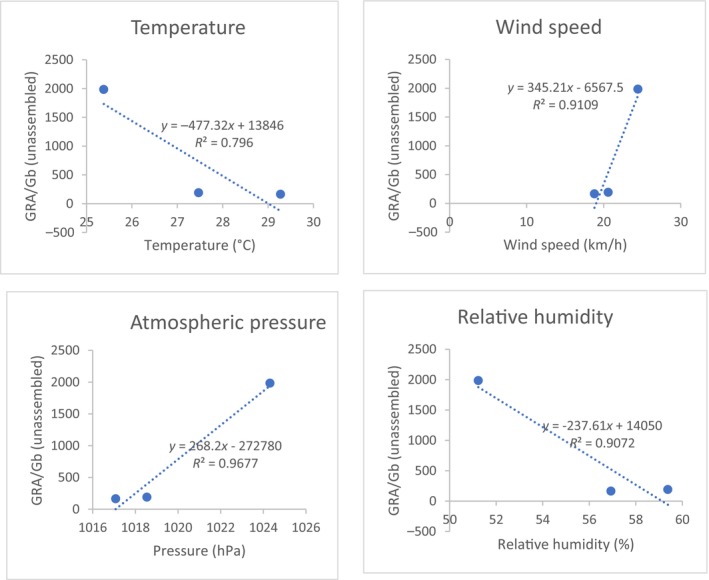
Comparison of ARG abundance in unassembled data in WWTP samples and different atmospheric parameters. The antibiotic resistance genes per unassembled Gb found in the air of WWTP out1, out2 and in situ were compared with the wind speed (km/h), temperature (°C), atmospheric pressure (hPa) and relative humidity (%). Atmospheric data provided by AVAMET. ARGs, antibiotic resistance genes; WWTP, wastewater treatment plant.

Various atmospheric parameters, such as temperature (Ravva et al., 2012) or relative humidity (Ouyang et al., 2020) could influence the concentration and distribution of certain ARGs in diverse ways. We compared those with our ARGs found in the unassembled data (Figure A3). Wind speed has been found to correlate with the shift of ARG in the spring season (Zhou et al., 2023). In our case, although we have a limited sampling size, wind speed correlated with a greater ARG abundance, probably because it helps with the ARG dispersion.

### Characterisation of resistomes and antibiotic‐resistant bacteria in airborne particles

**FIGURE 4:**
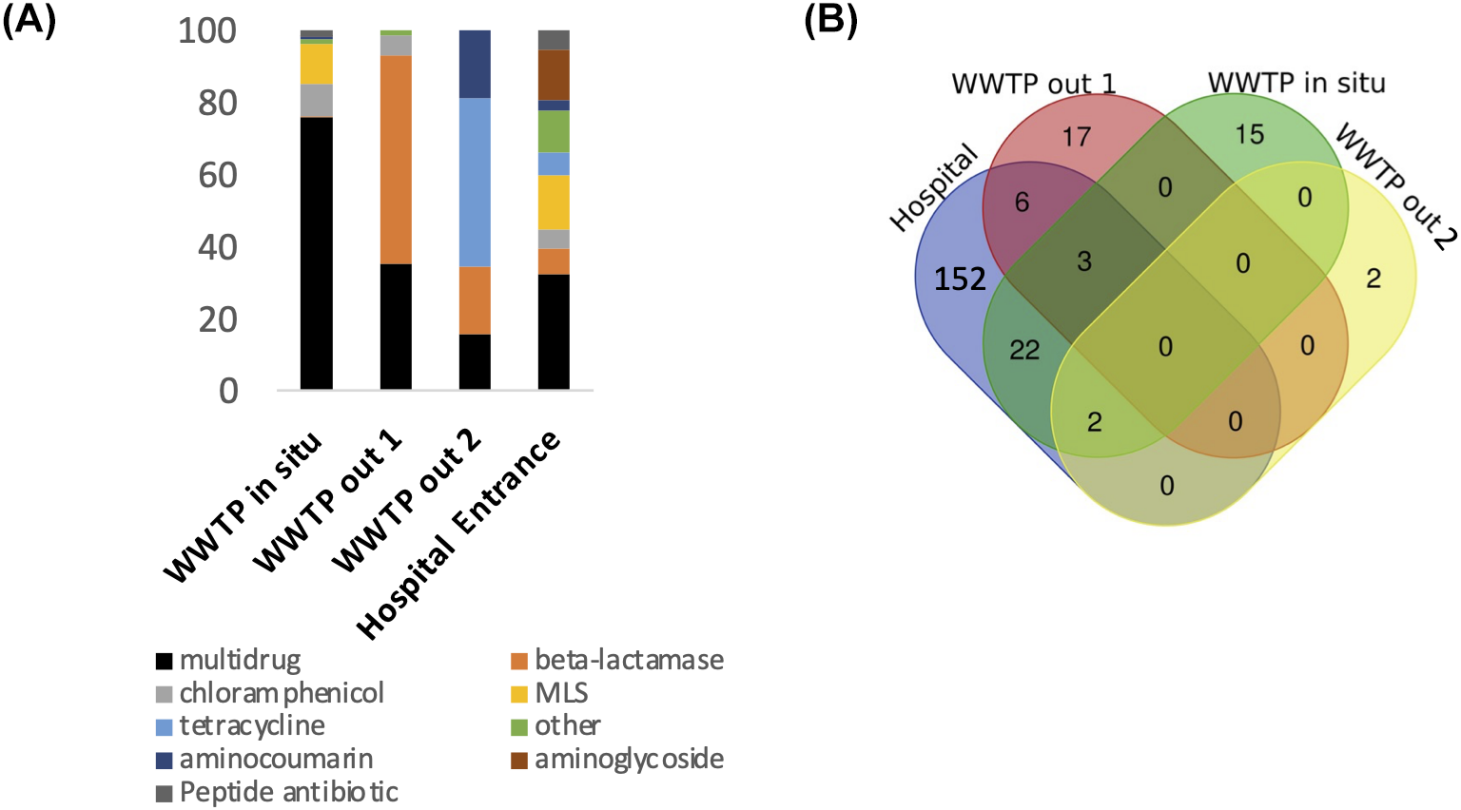
Characterisation of airborne resistomes. The ARGs found in the air sample were grouped according to the category of antibiotic they confer resistance to (A). Venn diagram showing shared and unique ARGs (90% id; unassembled data) found in the different air samples (B). ARGs, antibiotic resistance genes; WWTP, wastewater treatment plant.

The resistome analysis showed that most of the detected ARGs provided resistance to beta‐lactam antibiotics, chloramphenicol and tetracycline, among others (Figure 4A). Beta‐lactam and macrolide antibiotics are described as the most frequently used in hospital settings (Chang et al., 2019). In our study, we find that the predominant ARG category in the main entrance hospital sample is MLS, followed by aminoglycoside resistance (which is usually combined with beta‐lactams and fluoroquinolones for treating severe infections; Avent et al., 2011). In the third position are beta‐lactam antibiotics, suggesting that the antibiotic administered in the hospital may contribute to the resistance found in air (Zhou et al., 2021).

**FIGURE 5:**
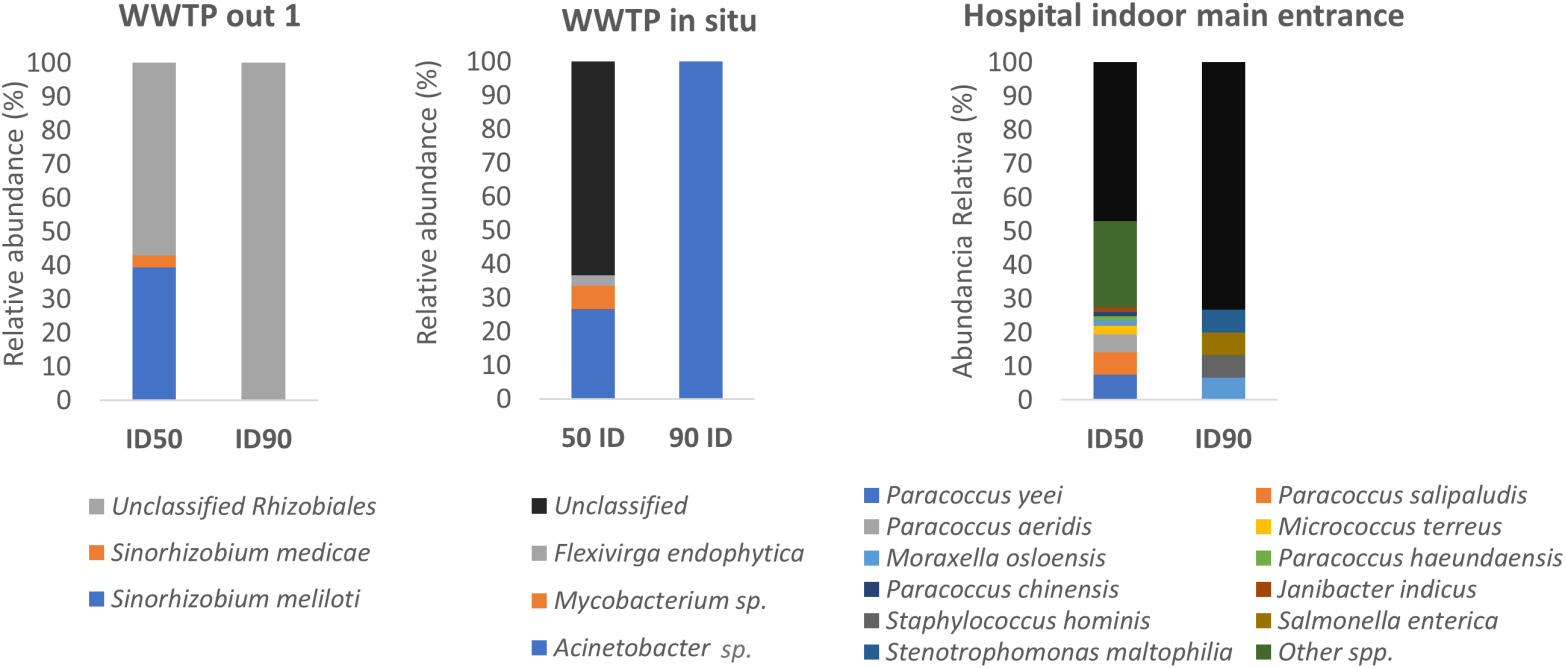
Main antibiotic resistance bacteria found in airborne particles. Unassembled data was metagenomically assembled and contigs were analysed for ARG detection and taxonomic identification according to genetic content. Data are shown in the relative abundance of detected contigs containing at least one ARG using two different thresholds, 50% and 90% of identity against ARG databases. Results for WWTP out 2 was not shown since no ARG were detected in the analysed contigs. ARGs, antibiotic resistance genes; WWTP, wastewater treatment plant.

It is worth noting that the highest richness of ARG types was obtained for the main hospital entrance with a total of nine different major categories of antibiotic resistance, followed by the in situ WWTP location (Figure 4A). A total of 152 different ARGs were found only at the Hospital's main entrance. No ARG was common and shared in all analysed samples, and only the multidrug efflux pump *acr*B, a widely studied transporter with a high capacity for transporting a vast range of substrates (including antibiotics) (Reygaert, 2018), was shared by two (hospital main entrance and WWTP in situ) out of the four analysed samples (Figure 4B; Table S3). Regarding the ARG airborne microbial vectors, in the main entrance of the hospital, multiple bacterial species were found carrying ARGs, such as *Moraxella, Staphylococcus, Micrococcus*, and so forth (Figure 5). For instance, we detected *Salmonella enterica subsp. enterica serovar Enteritidis*, a pathogen associated with food‐borne diseases (Fardsanei et al., 2017) with the ARGs *strB* and *APH*(*3″*)‐Ib (aminoglycoside resistance) and *Stenotrophomonas maltophilia*, an emerging global opportunistic pathogen that causes hospital‐acquired infections (Brooke, 2012), which transported the *sme*R gene (beta‐lactam and aminoglycoside antibiotic resistance) (Figure 5).

**FIGURE A4:**
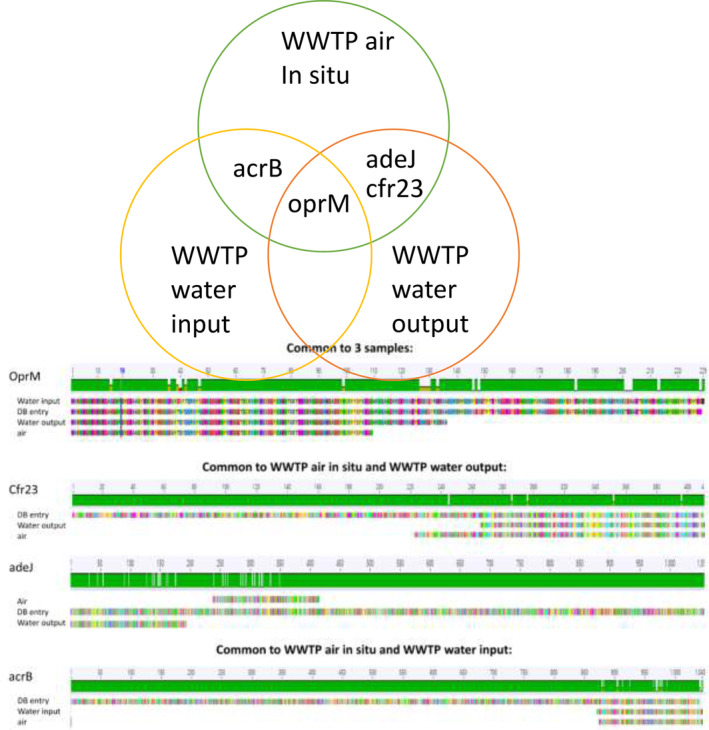
Common ARGs found in the air and water of the studied WWTP. All 4 ARGs found in the assembled data of the WWTP in situ (OprM, Cfr23, adeJ and acrB) were found in water samples taken from the same studied WWTP's waters (input, output or both; Panel A). Those ARG were aligned among them and with the ARG database entry (BD entry) that was used to classify them using the MUSCLE algorithm. The green bar indicates an amino acid identity of 100%, while the gaps indicate less or zero identity. The percentage of identity between the same ARGs (not including the ARG DB entry) in different samples varied from 91.2% to 100% for Oprm, cfr23 and acrB. The adeJ ARG was found in the air in situ sample and the WWTP output but both proteins did not align among them. ARGs, antibiotic resistance genes; WWTP, wastewater treatment plant.

Regarding the WWTP samples, we found ARGs in WWTP out 1 and in situ (Figure 3), while no ARGs were found in the WWTP out 2 assembled data. This sample WWTP out showed the lowest amount of assembled data (Table S1) despite a moderate‐high sequencing depth, emphasising the imperfections in metagenomic assembly (Lapidus & Korobeynikov, 2021). For the sample WWTP out 1, *Rhizobiales* and unclassified *Rhizobiales* (Figure 5), are commonly present in soil environments and potentially resistant to antibiotics due to their terrestrial origin, which hosts numerous antibiotic‐producing bacteria (Naamala et al., 2016), were detected carrying ARGs. For the WWTP in situ sample, common antibiotic‐resistant bacteria, such as *Acinetobacter*, were found. The contrasting differences in the type of ARGs and antibiotic‐resistant microbes found in those samples are not surprising because it is important to clarify that sample WWTP out 1 was taken in a spot where much of the wind during sampling was coming from the agriculture landscape was *Rhizobiales* are expected (Naamala et al., 2016), while the sample WWTP in situ was collected next to and above the WWTP biodigester, where *Acinetobacter* spp. were among the most abundant antibiotic‐resistant bacteria found in Alicante waters (Maestre‐Carballa et al., 2024). Therefore, in this sample, the air composition may be influenced by the bacterial composition of the wastewater (Han et al., 2019). All ARGs found in contigs of the WWTP air in situ sample (*n* = 4), were also found in the assembled data of the same WWTP's waters (input, output or both; ARG identity ≥90%) (Maestre‐Carballa et al., 2024). The identity percentage among those proteins ranged between 91.1% and 100% (Figure A4) for three ARGs but one (*adeJ*), whose proteins did not align among them. In addition, all contigs but one (WWTP output) were classified as *Actinobacter* by Kaiju analysis.

### Final considerations and limitations

Multiple broad‐range factors (i.e., changes in wind direction) affect the highly dynamic nature of the spatiotemporal distribution of airborne ARG (Li et al., 2016; Zhou et al., 2023). Despite our limited number of samples, our data show that the abundance and type of ARG were highly variable between them, even for those outdoor samples taken a few 100 m away from the WWTP site. The in situ WWTP outdoor sample showed the highest abundance, which might be explained because the sampling location was near the stirred tank bioreactor that emits numerous aerosols. Remarkably, and in good agreement with another recent survey (Zhou et al., 2023), in the indoor hospital sample, the amount and richness of ARGs in airborne particles was significantly high. Considering that a person breathes around 0.35 m^3^ of air per hour (Mack et al., 2019) and that we have obtained about ≈5 ARG hits per m^3^ and Gb of metagenome, data suggest that the thread of ARG dispersion throughout airborne particles should not be ignored, especially, for populated built environments. If we consider the fact that in our human body, the nares show one of the highest ARG loads (Liu et al., 2015; Maestre‐Carballa et al., 2022), then it becomes obvious that global ARG surveillance strategies should include as a standard, at least, the assessment of air quality and ARG monitoring in indoor environments.

In our study, we have used sequence similarity‐based searches with strict cut‐offs for detecting ARGs in metagenomics datasets to avoid false positives. Hits with amino acid identity ≥90% and bit‐score ≥ 70 against ARGs deposited in curated reference antibiotic resistance databases should be considered robust. These criteria have been used in all of our analyses except for Figure 5 which included the strict criteria and a more relaxed threshold (≥50% amino acid identity and bit‐score ≥ 70). This approach for detecting ARG has been widely used in previous publications (Chng et al., 2020; Lira et al., 2020; Maestre‐Carballa et al., 2019, 2022; Van Goethem et al., 2018). Likewise, our methodology can probably rule out some actual ARGs that display a score similarity below our thresholds. Nevertheless, as highlighted in previous surveys, a more rigorous threshold is preferred (Enault et al., 2017).

In conclusion, our metagenomic data from airborne particles revealed the detection of a wide array of ARGs and airborne antibiotic‐resistant bacteria including pathogens, potentially disseminated at high abundances per cubic meter of air, both in outdoor and indoor locations. Our metagenomic data suggest that the role of airborne viruses disseminating ARGs is likely negligent. Breathing polluted non‐ventilated air represents therefore a substantial threat to humans or animals that might aid in spreading ARGs that should be carefully considered within the One Health perspective.

## AUTHOR CONTRIBUTIONS

**Lucia Maestre‐Carballa:** Conceptualization (supporting); data curation (lead); formal analysis (lead); investigation (lead); methodology (lead); software (lead); visualization (lead); writing – original draft (lead); writing – review and editing (lead). **Vicente Navarro‐López:** Funding acquisition (lead); investigation (supporting); project administration (lead); resources (lead); writing – original draft (supporting); writing – review and editing (supporting). **Manuel Martinez‐Garcia:** Conceptualization (lead); data curation (supporting); formal analysis (supporting); funding acquisition (lead); investigation (supporting); methodology (supporting); project administration (lead); resources (lead); supervision (lead); validation (supporting); visualization (supporting); writing – original draft (equal); writing – review and editing (equal).

## CONFLICT OF INTEREST STATEMENT

The authors declare no conflicts of interest.

## Supporting information


**TABLE S1.** Shows number of ARG hits obtained by metagenomic approach.

**TABLE S2.** Taxonomic assignment of raw reads.

**TABLE S3.** Antibiotic resistance genes found in unassembled data.

## Data Availability

Sequencing data is openly available under BioProject PRJNA1029630: https://www.ncbi.nlm.nih.gov/bioproject/PRJNA1029630.
